# Examination of head *versus* body heading may help clarify the extent to which animal movement pathways are structured by environmental cues?

**DOI:** 10.1186/s40462-023-00432-y

**Published:** 2023-10-27

**Authors:** Richard M. Gunner, Rory P. Wilson, Mark D. Holton, Nigel C. Bennett, Abdulaziz N. Alagaili, Mads F. Bertelsen, Osama B. Mohammed, Tobias Wang, Paul R. Manger, Khairi Ismael, D. Michael Scantlebury

**Affiliations:** 1 Department for the Ecology of Animal Societies, Max Planck Institute of Animal Behavior, 78467 Konstanz, Germany; 2 Department of Biosciences, College of Science, Swansea University, Swansea, SA2 8PP Wales; 3 Mammal Research Institute, Department of Zoology and Entomology, University of Pretoria, Pretoria, 0002 South Africa; 4 Zoology Department, King Saud University, P. O. Box 2455, Riyadh, 11451 Saudi Arabia; 5 Copenhagen Zoo, Centre for Zoo and Wild Animal Health, Frederiksberg, Denmark; 6 KSU Mammals Research Chair, Zoology Department, King Saud University, P.O Box 2455, Riyadh, 11451 Saudi Arabia; 7 Zoophysiology, Department of Biology, Aarhus University, Aarhus, Denmark; 8 School of Anatomical Sciences, Faculty of Health Sciences, University of the Witwatersrand, Johannesburg, South Africa; 9 Prince Saud Al-Faisal Wildlife Research Center, National Center for Wildlife, Taif, Saudi Arabia; 10 School of Biological Sciences, Queen’s University Belfast, 19 Chlorine Gardens, Belfast, BT9 5DL UK

**Keywords:** Animal behaviour, Movement, Decision-making

## Abstract

Understanding the processes that determine how animals allocate time to space is a major challenge, although it is acknowledged that summed animal movement pathways over time must define space-time use. The critical question is then, what processes structure these pathways? Following the idea that turns within pathways might be based on environmentally determined decisions, we equipped Arabian oryx with head- and body-mounted tags to determine how they orientated their heads – which we posit is indicative of them assessing the environment – in relation to their movement paths, to investigate the role of environment scanning in path tortuosity. After simulating predators to verify that oryx look directly at objects of interest, we recorded that, during routine movement, > 60% of all turns in the animals’ paths, before being executed, were preceded by a change in head heading that was not immediately mirrored by the body heading: The path turn angle (as indicated by the body heading) correlated with a prior change in head heading (with head heading being mirrored by subsequent turns in the path) twenty-one times more than when path turns occurred due to the animals adopting a body heading that went in the opposite direction to the change in head heading. Although we could not determine what the objects of interest were, and therefore the proposed reasons for turning, we suggest that this reflects the use of cephalic senses to detect advantageous environmental features (e.g. food) or to detect detrimental features (e.g. predators). The results of our pilot study suggest how turns might emerge in animal pathways and we propose that examination of points of inflection in highly resolved animal paths could represent decisions in landscapes and their examination could enhance our understanding of how animal pathways are structured.

**Supplementary Information:**

The online version contains supplementary material available at 10.1186/s40462-023-00432-y.

## Introduction

The study of how animals distribute themselves in space – how life relates to the environment - is one of the pillars in ecology [1]. To elucidate this among vertebrates, data on animal location (for definition of this and other terms see Supplementary text) are increasingly being gathered using animal-attached tags [2–4] which have now progressed to systems that provide high accuracy (typically to within a few metres) [5], although most are deployed to be temporally irresolute (generally giving location information at hourly intervals or less [6–8]).

The high spatial accuracy of new systems though, has resulted in researchers using such location data to populate models that describe animal movement, the process that leads animals to occupy space in the ways they do. These models are based on changes in animal locations between fixes, with these ‘step lengths’ being used as descriptors, although they are inevitably affected by the temporal resolution of the fixes. Models for movement patterns include, for example; random walk [9], correlated random walk [10, 11] and Lévy walk [12].

Pyke [13] argued that the use of irresolute animal location data made it difficult to relate movement paths to sensory systems that might inform decisions on how to move [14], most particularly if the scales at which animals make movement decisions (presumed based primarily on sensory information [cf. 14]) are smaller than the resolution of the approach used to describe them. Rather, Pyke purported that, to understand the movements of organisms, work should have the appropriate temporal and spatial resolution to focus on decision-making processes that affect movement [15].

In their movement ecology paradigm for unifying organismal movement research, Nathan et al. [14] regard external factors as one of their four primary mechanistic aspects affecting how animals move. Thus, movement decisions – specifically where, when and why animals have turns in their movement paths [16] - are the fundamental processes that translate into animal space use because turns give movement paths their structure. Indeed, this perhaps explains why initial, recent work looks at turn points in finely resolved animal paths [16, 17], something which also dovetails with classic animal behaviour choice studies such as those examining decisions taken in diverging tunnels in mazes [18].

We suggest that a reasonable schema for considering how animals modify their pathways from a default ‘straight line path’ starts with some elicitor for turning behaviour which can then affect whether a turn should take place and the extent of the turn. The structure of the path, and the eventual space use by the animal follows as a consequence of this. As such, the turn elicitor should be key to understanding movement paths and space use in general.

To examine this, we use a new spatially and temporally highly resolute approach to try to identify how and when turns in animal paths occur [cf. 19]. For this, we conducted a pilot study where we used direction- and location-determining systems on the heads and bodies of semi-captive animals (Arabian Oryx, *Oryx leucoryx*) to elucidate how head ‘behaviour’ related to the subsequent movement path. Head behaviour is important because sensory systems are generally concentrated in the head [20, 21], this being the leading body part in normal movement, informing about conditions ahead. As a consequence, we reasoned that head heading should be linked to body heading during movement and that examination of both should elucidate mechanisms behind changes in animal paths. Specifically, we had two hypotheses; (i) that over scales of minutes, animals subjected to an increasingly intense stimulus would first react to it by orientating their heads to assess the stimulus before, eventually, moving their body in response and (ii) that, over time scales of seconds, changes in head heading that occurred during normal locomotion, would precede changes in body heading, ultimately leading to turn points in the animal paths. We appreciate that animals responding in their movement patterns to external stimuli in this way is an oversimplification since it has been noted that both memory and internal ‘state’, such as blood sugar level, can also elicit changes in animal movement paths [14]. Nonetheless, we believe that response to external stimuli perceived by animal sensors may be a major reason why animals change the form of their movement paths and use this as a prime consideration in our treatise below.

## Methods

Six Arabian oryx, from a herd of 12 semi-captive animals kept within a 0.5 × 0.5 km natural enclosure within the Imam Saud bin Abdulaziz Royal Nature Reserve (previously called Mahazat as-Sayd), a protected area located in west-central Saudi Arabia (41.58, 22.34), were equipped with head- and body-mounted tags (Daily Diary – standard model; http://www.wildbytetechnologies.com/tags.html) that recorded tri-axial acceleration at 40 Hz and tri-axial magnetic field intensity at 13 Hz using a real-time clock base [22]. The head-mounted tags were glued between the horns while the body-mounted units were attached to a collar and weighted to hang ventrally. The collar also had a dorsally mounted GPS, set to determine location once every 15 min. Although the real time clocks in the devices were reported to have maximum drift of no more than a few seconds per month, all units were calibrated to synchronize time properly. This was done at the beginning and end of deployments by taping all units to a tray and subjecting them to sharp movements that could be defined with respect to time by the accelerometers so that any drift over the full deployment period could be corrected linearly. Following this, checks on when movement was initiated by animals after long periods of rest in periods up to day 3 showed that the timing of the acceleration signatures defining the onset of movement from the head- and body-mounted tags were indistinguishable. Hard and soft iron distortions were corrected according to Gunner et al. [23].

The animals were then allowed to roam freely within their enclosure for 7 days. On day 6, two predator simulation exercises took place that were video-recorded. In the first, a single person approached the (closely associated) herd from 685 m to ca. >10 m over 16 min, until the animals reacted by moving away. The person then returned to the start location and, 12 min later, together with another person, approached the herd in a similar manner, but this time using a pincer movement where the two people approached from different directions, until the herd again reacted by moving away (Animation S1).

On day 3, we selected a single period of ca. 5 h of behaviour for each individual, during which the focal animals were predominantly moving, as indicated by Vectorial sum of the Dynamic Body Acceleration [24] having values > 0.1 *g.* These were judged to be representative of ‘normal movement behaviours’ for this species.

After recovery of the tags, the individual head- and body-headings from all animals for all periods were determined using methods described in Gunner et al. [23] as well as times when significant changes in either metric occurred (Fig. S1) and derivation of the animal movement paths. The fine-scale movement of all animals was determined using verified location-enhanced dead-reckoning [23] (see Supplementary text for further details). ‘Significant turns’ in the head- and body heading were identified using the turning-point algorithm described by Potts et al. [17]. The algorithm looks for changes in the body heading by sliding a small window (w = 40 consecutive points (over 1 s) and used here) across the path and observes the squared circular standard deviation (SCSD) across the window. Spikes in the SCSD indicates a turn in the path and candidate turns are filtered according to whether they achieved a threshold turn angle (thresh = 30^o^ used here).

**Fig. 1:**
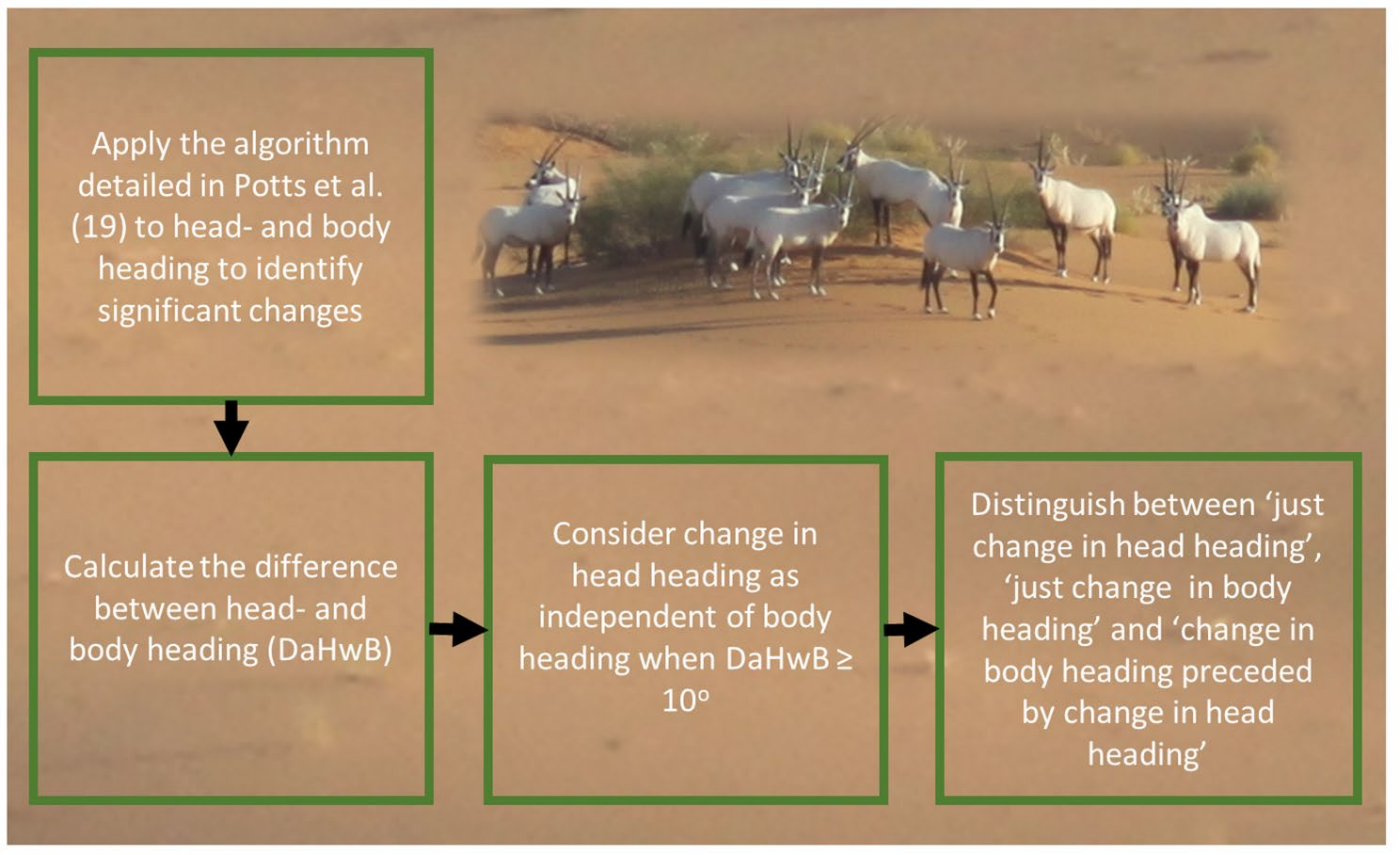
Schematic diagram of the process used to calculate changes in head- and body headings and whether they were significant

Three basic categories were recognised based on how the head heading changed with respect to the body heading during movement and, to facilitate description of this, we termed the difference in the degree of alignment of the head with respect to the body (in the horizontal plane) ‘DaHwB’ (Supplementary text). The three different scenarios that we recognised were: (i) **changes in just head heading**, where the head changed significantly but the body did not [DaHwB increased]; (ii) **changes in just body heading**, where the body heading changed significantly in a manner that was mirrored in time and extent by the head heading (so that DaHwB effectively did not change significantly from zero); and (iii) **changes head heading followed by changes in body heading**, where an independent change in head heading (Fig. 1) was followed by a change in body heading within 3.5 s [DaHwB first increased and then decreased within 3.5 s].

**Fig. 2:**
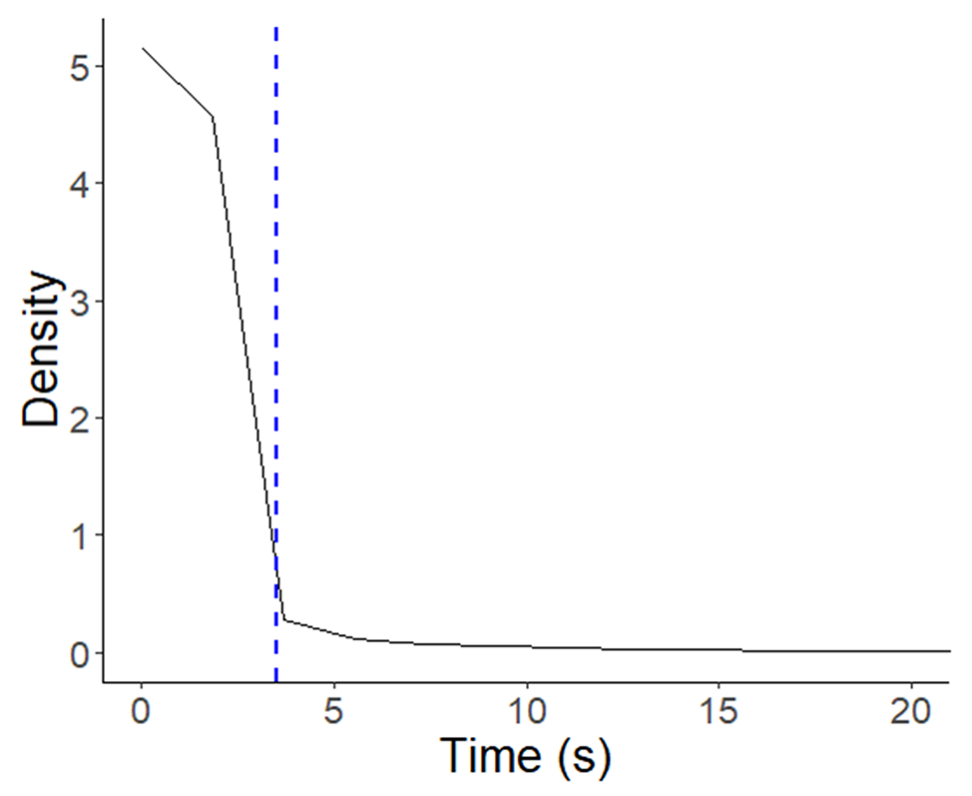
Summary frequency distribution of the time elapsed between ‘head-’ and subsequent turns in the movement paths for all oryx (manifest by changes in body heading). The blue vertical dashed line at the point of inflection corresponds to 3.5 s, the threshold time window, less than which changes in body heading were considered to be associated with the action of the head

The 3.5 s threshold for the association between head- and body-turns was based on density *versus* time plots for changes in head heading and the next change in head heading resulting in a turn in the movement path (Fig. 2).

## Results

### Reaction to threats

**Fig. 3:**
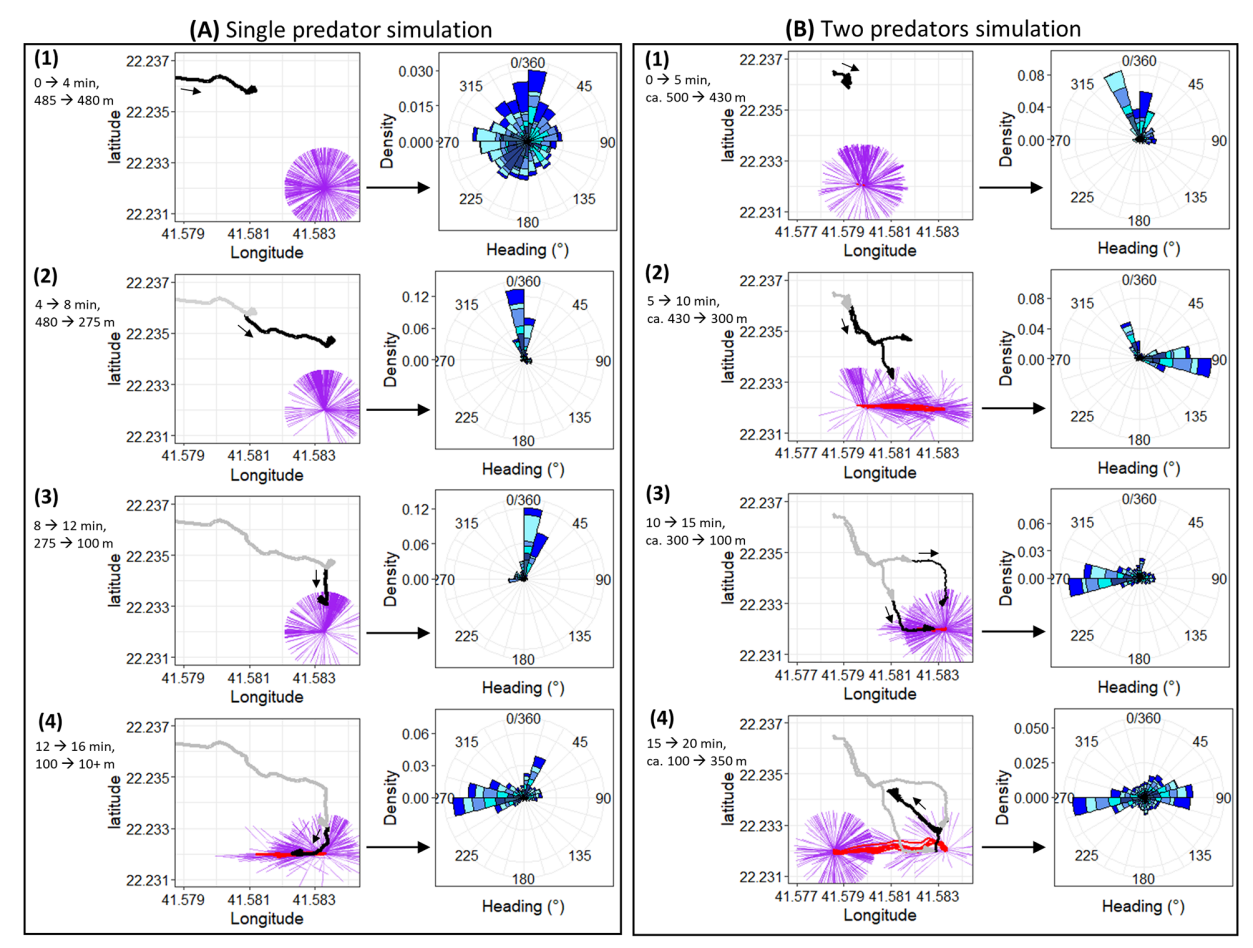
(**A**) Approach by people to 6 oryx within a ca. 0.5 × 0.5 km pen by walking (starting top left) in a (**A**) single-, then (12 min later) (**B**) double predator simulation. The person/people approaching is shown in black with timings and distances to the left while the locations of the oryx are shown in red, with head headings depicted by straight ‘hairs’. The predominant head heading for the different individuals (different colours) for each sector is shown to the right. Note how in (**A**) the single predator simulation, the oryx initially have head headings in a predominantly circular pattern (panel A1), but rapidly (at distances of < 480 m) fixate on the human (pane A2) until distances of < 275 m when some changes in head heading (scanning) appears to concentrate on an escape path (pane A3). Finally, at < 100 m, the animals concentrate their attention on the escape path, with less attention maintained on the human (pane A4). In (**B**), the two predators simulation, similar patterns occur, including the animals apparently looking for an escape route (pane B2) but, as the humans approach to < 300 m, with attention seemingly focused on the closest person (pane B3)

When we simulated potential predators (humans) advancing on a group of 12 oryx, 6 of which were equipped with body and head tracking technology, we determined that, as the stimulus approached, all animals preferentially changes their head headings so that they faced the threat (Fig. 3), indicating that the direction that the head heading took is an important correlate of stimuli of interest.

Specifically, when one person approached the group at distances of up to ca. 500 m, head heading was distributed relatively evenly (Fig. 3A1). With distance then decreasing to 275 m, all animals directed their heads towards the approaching person (Fig. 3A2), while at distances of between 275 and 100 m, the head heading of the animals became bimodal, predominantly looking at the approaching person, but also apparently with headings seeming to indicate the animals fixating on an escape route although all oryx remained stationary (Fig. 3A3). Finally, at shorter human-oryx distances (less than 100 m), the animals had a head heading that resulted in their heads being pointed predominantly towards the escape route as they moved away (Fig. 3A4) (Animation S1). The group approached by two people from different directions showed similar head heading variability (Fig. 3B1, B2, B4), but notably animals prioritized the closer person (Fig. 3B3).

A more detailed examination of the single predator simulation (Fig. 3A) indicated an increase in the number and magnitude of heading changes as the human approached. Specifically, these related to; (i) just head heading and (ii) body heading changes preceded by changes in head heading. This contrasted the uninformed changes exclusively in body heading (see supplementary text; Fig. S2).

### Non-threatening environments

**Fig. 4:**
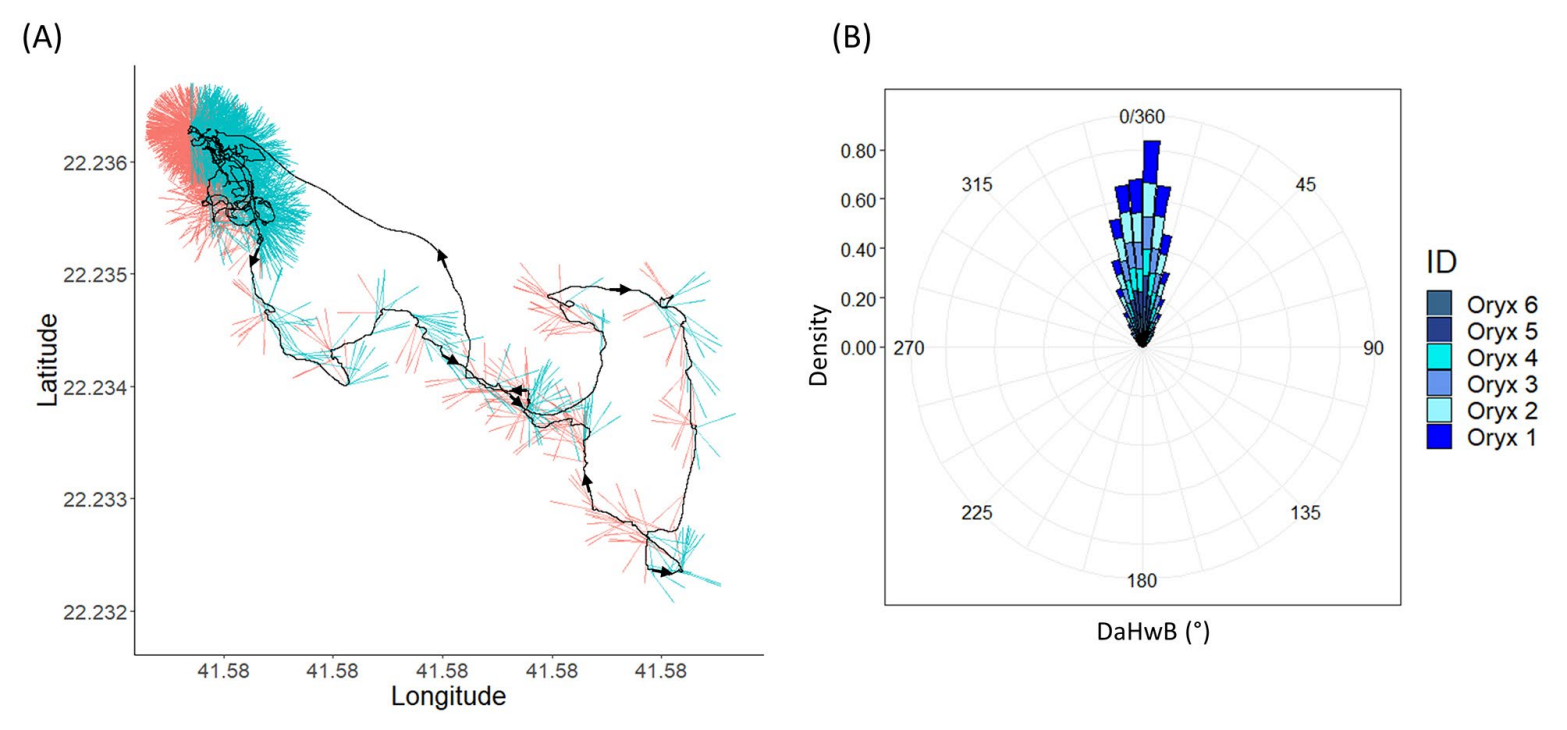
DaHwB (the angular difference between the heading of the head and the body) during routine movement. (**A**) Example of an oryx head heading (depicted by ‘hairs’ emanating from the animal movement path – black line) when it deviated from body heading (DaHwB - with red hairs showing head heading being anticlockwise [left] of the body heading and blue hairs showing the reverse) and was followed by a change in body heading within 3.5 s (see Fig. 2) resulting in a turn in the path – note the differing scales of path tortuosity over the length of the track. (**B**) Density distribution of head DaHwB of the 6 individual oryx (colour-coded in blue), illustrating that, although changes in head heading with respect to body heading may be substantial during oryx movement (see A), the head and body are well aligned for most of the time

In non-threatening environments, animals exhibited complex paths as they moved, with head heading periodically differing substantially from body heading (Fig. 4A) even though, overall, there was good concurrence between both (Fig. 4B).

**Fig. 5:**
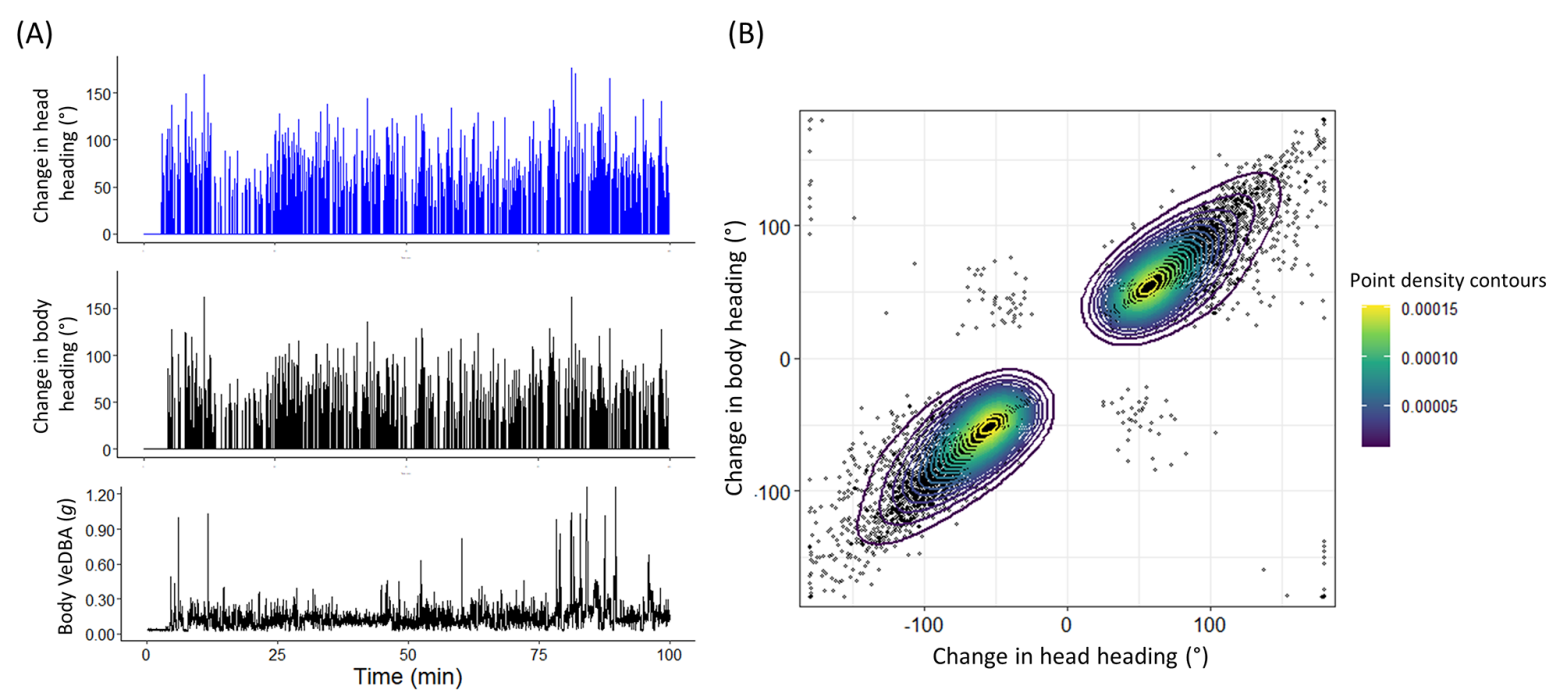
The relationship between head- and body heading. (**A**) 100 min of an individual oryx movement data showing the incidence and extent of change in head heading in relation to the body heading. The lower panel shows the Vectorial Dynamic Body Acceleration, a proxy for movement speed. (**B**) Data on all changes in head heading from 6 oryx, each over a mean of 5.15 animal hours (SD 0.15), in relation to the extent of the subsequent change in body heading showing the correlation between them (see text for details). This representation also has the area divided into four quadrats to help understand what each point means. Data in the bottom left and top right quadrats constitute cases where the head heading changes are in the same direction as the body heading whereas data in the top left and bottom right quadrats constitute cases where the head heading changes are in a direction opposite to the body heading. Note that a very small percentage of points have the head looking back over the animals’ shoulders (with a DaHwB of 180°). These values are due to the animals indeed looking back over their shoulders but may have more error in heading because at this time the collar may be pressed against the body, displacing it from its normal position on the neck to some extent

Where significant deviations in DaHwB were followed (within 3.5 s – Fig. 2) by changes in body heading (Fig. 5A), the extent of the change in head heading and preceding change in body heading were highly correlated (circular-circular correlation: rho 0.93, p < 0.001; Fig. 5B).

**Fig. 6:**
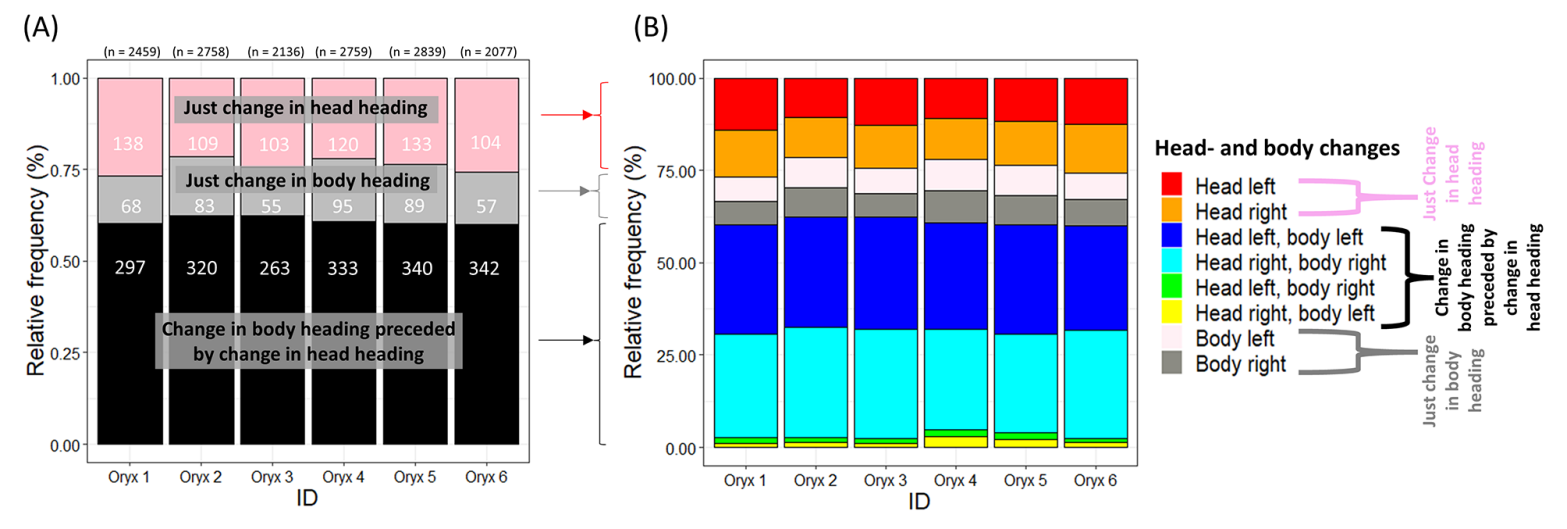
Relative incidences of changes in head- and body heading in 6 oryx during a mean of 5.15 h (SD 0.15) of routine movement per individual. (**A**) According to whether; only the heading of the head changed (pink), only the heading of the body changed (grey) or changes in the head heading preceded a change in body heading (black) [total number of instances given in brackets, and the number of heading changes per hour given in white text]. (**B**) Details of changes in body heading without changes in head heading (whether anticlockwise or clockwise [to the left or right] of the heading adopted by the body), changes in head heading (whether to the left or right of the body heading) and, in the case of changes in head heading followed by changes in body heading, their relation to the subsequent changes in body heading (whether left or right) for individual oryx. Note the roughly equal number of left and right heading changes for both bodies and heads, whether linked or not, but that the incidence of cis-head followed by changes in body heading (head heading moved left of the body heading before the body heading moved left or the head heading moved to the right of the body heading before the body heading moved right) greatly exceeded the reverse situation

Generally, the oryx; (1) changed their body heading following a significant change in head heading in 61.1% (IQR 1.7) [IQR: Interquartile range refers to the difference between the upper and lower quartiles of relative frequencies collated for each individual] of all head/body heading changes, (2) exhibited ‘just changes in head heading’ in 24.0% (IQR 3.1) of cases, and (3) engaged in ‘just changes in body heading’ in 14.9% (IQR 2.8) of cases (Fig. 6A). ‘Just changes in head heading’, ‘just changes in body heading’, and ‘changes in head heading followed by changes in body heading’ were executed in equal proportions to the left and right (Fig. 6B). Notably, changes in head heading then changes in body heading in the same direction (head heading moved anticlockwise [left] then the body moved anticlockwise [left] and head ‘right’ then body ‘right’ – cis-turns) were made a mean of 21.1 times (IQR 7.5) more often than opposing changes in head heading then body changes in heading (head ‘left’ then body ‘right’ and head ‘right’ then body ‘left’ – trans-turns) (Fig. 4B). Conversely, although changes in head heading did occur (within 3.5 s) following changes in body heading, these instances were relatively uncommon, comprising only 36% of all head heading changes (of which, 77% were ‘informing’ changes for the subsequent body heading change). In contrast, changes in head heading that preceded changes in body heading represented 72% of all head heading changes. Changes in head heading that occurred after a body heading change constituted only 49% of the total body heading changes that were preceded by a head heading change.

## Discussion

Although the centrality of animal senses for informing animal behaviour is acknowledged within the literature [25], the link between what animals perceive and how they choose to move is poorly developed. Consideration of this may enhance our ability to determine the things that might affect animal movement and strengthen our capability to model pathways and animal area use although, technically, this approach is subject to appreciable challenges. Our predator simulation experiment demonstrates the importance of head heading for oryx as they inspect the environment [26], which we attribute to them gathering information of relevance to decide whether to change or maintain their current path [cf. 27, 28]. Importantly, the highly correlated changes in oryx head- and body headings strongly suggests that the direction of travel is largely informed by head movement [cf. 29]. In the case of the simulated predator(s), the head heading ultimately led to animals adopting an opposite body heading as they moved away. Within this context, although we could not identify the specific features of the environment that might act as turn elicitors within their normal movement pathways, we suggest that the most common head/body interaction, cis-head and body heading, occurs because animals have identified an object or area of interest during changes in head heading after which the body heading changes to move the animal towards this site [30]. The relative paucity of trans-head and body heading cases is presumably related to the safety of the oryx compound where stressors, such as predators, are essentially absent. Changes in head heading without a correlated change in body heading can be attributed to monitoring of the environment [26], within which the movements of conspecifics are included, without finding any beneficial or detrimental aspects that might initiate a path change. The incidence of changes in body heading without prior changes in head heading may be due to sub-threshold head DaHwB informing changes in body heading (including capitalizing on the extended lateral visual field of artiodactyls that is presumably present in the Arabian oryx [31]) or animals responding to changes in body heading made by conspecifics [32]. Given the high incidence of links between DaHwB and change in body heading (Fig. 5), it seems unlikely that most of these ‘un-informed’ changes in body heading are the result of random decisions [33].

**Fig. 7:**
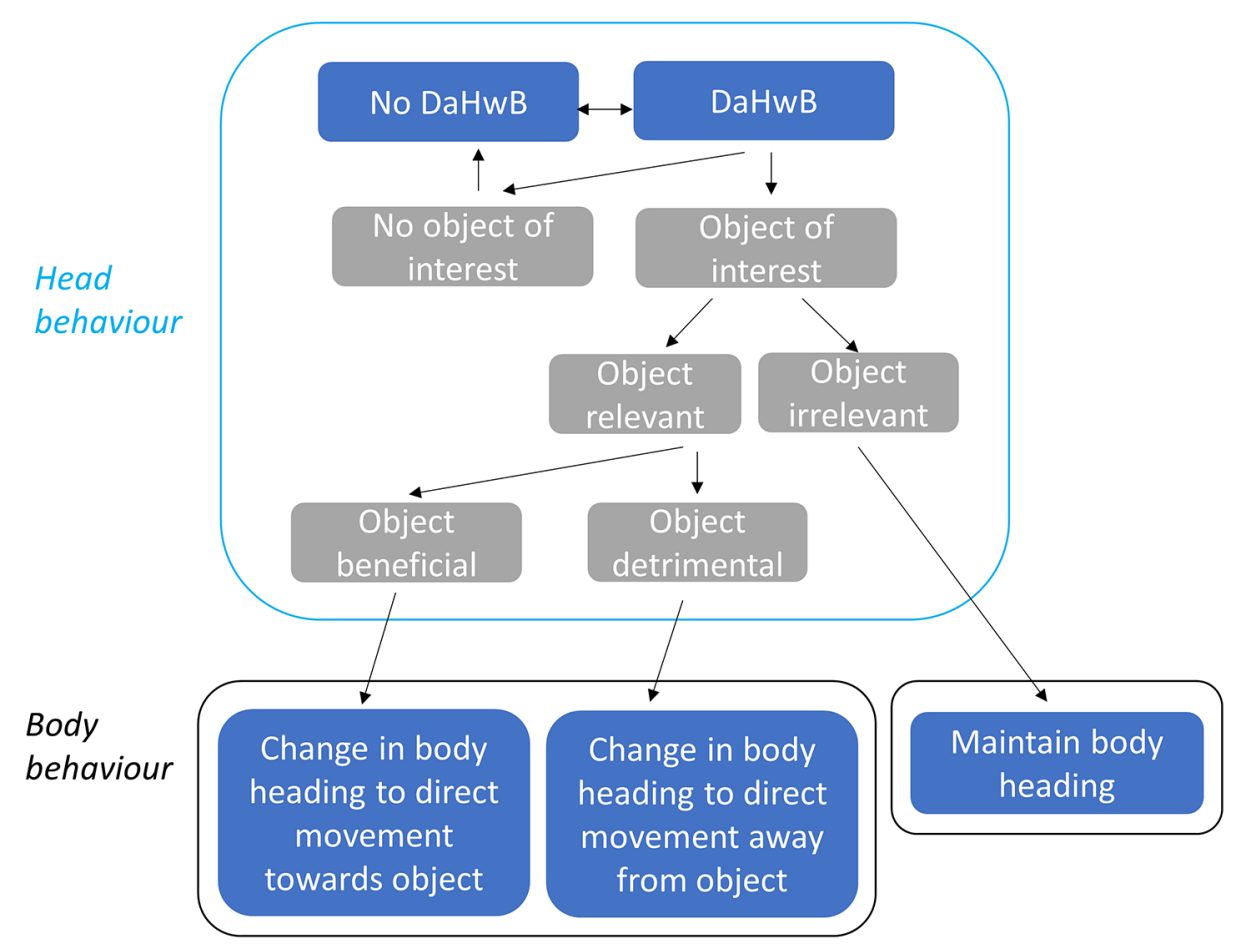
Proposed links for changing DaHwB (the difference between head heading and body heading) and associated subsequent body heading during movement

Rather than ascribing a high degree of probabilistic or random elements within animal pathways, based on our results, we propose the following schema for helping explain animal movement paths: Notwithstanding that animals may change heading as a result of memory and/or internal state (14 - *see earlier*), moving animals engage in environmental scanning to inform themselves of features that are relevant to them [21]. Finding no cues relevant to navigation based on memory or changes caused by memory per se as well as no other relevant cues, a straight-line path will be maintained [16]. Where relevant environmental features are identified, animals will change body heading which directs them towards features identified as beneficial (e.g. food or proximity to conspecifics) and directs them away from detrimental features (e.g. a difficult energy landscape or threats from predation) [28] (Fig. 7).

This schema specifically implies that there may be less randomness in the step lengths and turn angles in animal pathways than previously thought. Rather, the mechanistic causes and functional motivations behind the processes that lead to particular movement types (as exemplified in a suite of powerful movement models [e.g. 34, 35–39]) could be substantially, and perhaps primarily, governed by informed decision-making. We suggest that the use of high resolution animal movement data should help to identify turns in animal pathways [17] and, according to our proposed schema, examination of the frequency and extent of turns should help identify decision points [16]. If combined with DaHwB data, this may even facilitate determination of whether the turns are towards perceived benefits or away from perceived detriment. Indeed, it may even be possible to examine DaHwB with respect to subsequent ‘informed’ body headings to populate a tracked animal’s environment with objects/issues of interest while the interplay of positive and negative reactions may help us to determine the density of beneficial and detrimental objects. This should allow the quantification of particular environments in terms of costs and benefits for the animal. For example, we expect hungry animals in landscapes of high food density to exhibit high frequency head scans and a high incidence of ‘attractant’ turns in the movement pathways – effectively resulting in area-restricted search (ARS) behaviour [cf. 40, 41]. Otherwise, animals, particularly herbivores which operate in high food density areas, might search for food less when it is plentiful, particularly if searching has a high cost. In contrast, detrimental landscapes (e.g. “a landscape of fear” [42] or heterogenous energy landscapes [43]) should be typified by high frequency scans [44] and a high incidence of ‘avoidance’ turns. Other modulators of animal pathways may be more opaque, such as landmark salience in spatial navigation [45]. Overall though, examination of the density of identified head heading changes relates to body heading with respect to space might help define landscapes of fear, energy landscapes or accident landscapes. 

## Conclusions

Overall, our work with Arabian oryx suggests that the identification of turns within highly resolved animal movement paths may help point to movement elicitors which may vary in density across space, although the issue of determining precisely what these elicitors are, will be a major challenge. Importantly though, the combination of two sensor systems, one on the head and one on the body, should allow workers to investigate how scanning can lead, or not, to movement patterns and paths.

### Limitations of the study

The sample size for this study was relatively small and only concerned one species in a semi-captive setting that limited behavioural options for the oryx. Further, no information on food resources were sampled which would have supplemented reported trends in the direction of animal movement. All this makes our work more of a pilot study in the area which attempts to link environmental cues mechanistically to animal pathways. Nonetheless, we speculate that this proposed schema derived from Arabian oryx should be a useful starting point for understanding the decision rules underlying the incidence of inflection points in other species’ movement paths although we recognise that more experiments are required across a range of species to justify this. Lastly, properly controlling for the effect of eye movement within the eye orbits, something that is fairly minimal in ungulates anyway, is difficult and we did not attempt it. Its effect, if there is an effect, might be to result in diminished change in head heading.

### Electronic supplementary material

Below is the link to the electronic supplementary material.

Supplementary Material 1

Supplementary Material 2

## Data Availability

The IMU Oryx datasets have been submitted to Dryad repository (10.5061/dryad.c59zw3rc1). The Dryad repository also contains an example R script that follows the steps outlined in Fig. 1, to detect ‘significant’ changes in body and head headings.
