# Supplementary material for: Examination of head versus body heading may help clarify the extent to which animal movement pathways are structured by environmental cues?
Source: Mov Ecol. 2023 Oct 27;11:71. doi: 10.1186/s40462-023-00432-y (PMC10612247; doi:10.1186/s40462-023-00432-y)
Supplement: Supplementary file 1 — Supplementary Material 1 [file 40462_2023_432_MOESM1_ESM.docx]

**Supplementary Materials for:**

**Examination of head versus body heading may help clarify the extent to which animal movement pathways are structured by environmental cues?**

Richard M. Gunner*, Rory P. Wilson*, Mark D. Holton, Nigel C. Bennett, Abdulaziz N. Alagaili, Mads F. Bertelsen, Osama B. Mohammed, Tobias Wang, Paul R. Manger Khairi Ismael, D. Michael Scantlebury

Corresponding authors – Richard M. Gunner – richard.m.g@hotmail.com, tel; +44 7715 921393; Rory P. Wilson – r.p.wilson@swansea.ac.uk, tel +44 1792 295376; D. Michael Scantlebury – email m.scantlebury@qub.ac.uk, tel +44 28 9097 2278.

* equal contributors

**This file includes:**

Supplementary text

References (1-4)

Figures S1-S2

**Other Supplementary Materials for this manuscript include the following:**

Animation S1

**Supplementary text**

*Definitions:*

**Heading** - In 2D, the azimuth, whereby the azimuth is the difference in angle between the point of interest directly ahead of the oryx (head or body) and true North, measured clockwise around the observer’s horizon.

**Location** – a point in space defined by Cartesian coordinates (x, y in 2D space and x,y,z in 3D space).

**Path/pathway** – An ordered series of locations with no time stamp.

**DaHwB** – degree of alignment of the head with respect to the body (in the horizontal plane). A DaHwB of 0⁰ indicates that the head and body headings are identical.

*Extended technical descriptions of the Materials and methods*

The study took place at the Imam Saud bin Abdulaziz Royal Nature Reserve (previously called Mahazat as-Sayd), a large protected area in west-central Saudi Arabia (28°15’ N, 41°40’E). Animal were sedated using a Dan-Inject dart gun (Daninject, Børkop, Denmark) with etorphine hydrochloride (Captivon 98, Wildlife Pharmaceuticals Ltd., White River, South Africa; 19 µg/kg), ketamine (Ketaminol Vet., MDS Animal Health, Intervet International B. V., Boxmeer, The Netherlands; 0.3 mg/kg), midazolam (Midazolam, Wildlife Pharmaceuticals Ltd., White River, South Africa; 0.13 mg/kg), and medetomidine (Zalopine 10 mg/mL, Orion Pharma, Espoo, Finland; 5 µg/kg). Full details of capture and animal handling are described elsewhere (*1, 2*).

The head-mounted tags were glued between the horns using a mixture of quick-set epoxy resin and cyanoacrylate “superglue” which stuck to the fur within approximately 30 s. Tags were then removed once oryx were recaptured by peeling them off – they were easy to remove as they were stuck to the hair, not the skin of the animals. Body-mounted daily diary units were attached to a nylon livestock collar and attached to a c. 200g weight so that they hung ventrally. Global positioning system (GPS) data loggers (i-gotU GT-120; Mobile Action Technology, Inc., Taiwan) were attached dorsally to the collar so that they were in good view of the sky and configured to record a locational fix once every 15 min.

*Determination of animal location*

The fine-scale movement of all animals was determined using verified location-enhanced dead-reckoning as detailed by Gunner et al (*3*). Briefly, this involved deriving speed estimates from the Vectorial sum of the Dynamic Body Acceleration (VeDBA) (*4*) and converting them to a distance coefficient ($q$) (eqns 6-7). Coordinates are then advanced using $q$ values in conjunction with body heading ($h$) (see above) (eqns 8-9).

$s=\left( VeDBA \bullet m \right)+c,$ (6)

$q=\frac{s \bullet TD}{R},$ (7)

${Lat}_{i}=asin\left( \sin{(Lat}_{0})\bullet\cos(q)+\cos{(Lat}_{0})\bullet\sin(q)\bullet\cos(h) \right)$, (8)

${Lon}_{i}={Lon}_{0}+atan2\left( \left( \sin(h)\bullet\sin(q)\bullet\cos{(Lat}_{0}) \right), \left( \cos(q)- \sin({Lat}_{0})\bullet\sin({Lat}_{i} \right)) \right),$ (9)

where, $s$ refers to speed (m/s), $TD$ refers to the time difference between values (s), $R$ is the approximate radius of the earth (~6378137 m) and ${Lat}_{0}$,${Lat}_{i}$ and ${Lon}_{0}$,${Lon}_{i}$ are the previous and present latitude and longitude coordinates, respectively. Note that the coordinates and heading are supplied in unit radians. To correct for drift between verified locations (VLs - GPS fixes in this case), the (Haversine) distance and heading between consecutive VLs that are used to correct, and the consecutive (time-matched) dead-reckoned locations were calculated. The distance between each VL was divided by the distance between the corresponding dead-reckoned locations to provide a distance correction factor which was multiplied to all intermediate $q$ values. The heading between each VL was subtracted by the heading between the corresponding dead-reckoned locations to provide a heading correction factor which was added to all intermediate $h$ values. After the correction factors had been applied, eqns 9-8 were repeated and this process continued iteratively, until the dead-reckoned path segments adhered ‘exactly’ to the corresponding ground-truthed locations.

*Statistical analysis relating to Fig. S2*

Three Wilcoxon rank-sum tests were used to compare the median values of absolute changes in heading between the first and second halves of the single predator simulation (respectively, 0-8 min, predator proximity *ca.* 685 🡪 275 m, and 8-16 min, predator proximity ca. 275 🡪 10 m; *cf.* Fig. 3A). These comparisons were conducted separately for significant changes in: i) just body heading, ii) just head heading, and iii) body heading preceded by changes in head heading. The tests revealed a statistically significant difference between the two phases when it came to ii) just changes in head heading (W = 4448.5, p < 0.01) and iii) changes in body heading preceded by changes in head heading (W = 10160, p < 0.01), but not for i) just changes in body heading (W = 934, p = 0.66). Considering that the magnitude and frequency of heading changes - exclusive to just head heading, and body heading preceded by changes in head heading - escalated as the human neared, in contrast to uninformed changes solely in body heading, it suggests that cephalic sensing plays a pivotal role in decision-making and the subsequent execution of those decisions.

**References**

1. M. F. Bertelsen *et al.*, The hairy lizard: heterothermia affects anaesthetic requirements in the Arabian oryx (Oryx leucoryx). *Veterinary anaesthesia and analgesia* 44, 899-904 (2017).

2. J. G. Davimes *et al.*, Arabian oryx (Oryx leucoryx) respond to increased ambient temperatures with a seasonal shift in the timing of their daily inactivity patterns. *Journal of biological rhythms* 31, 365-374 (2016).

3. R. M. Gunner *et al.*, Dead-reckoning animal movements in R: a reappraisal using Gundog.Tracks. *Animal Biotelemetry* 9, 23 (2021).

4. R. P. Wilson *et al.*, Estimates for energy expenditure in free-living animals using acceleration proxies: A reappraisal. *Journal of Animal Ecology* 89, 161-172 (2020).


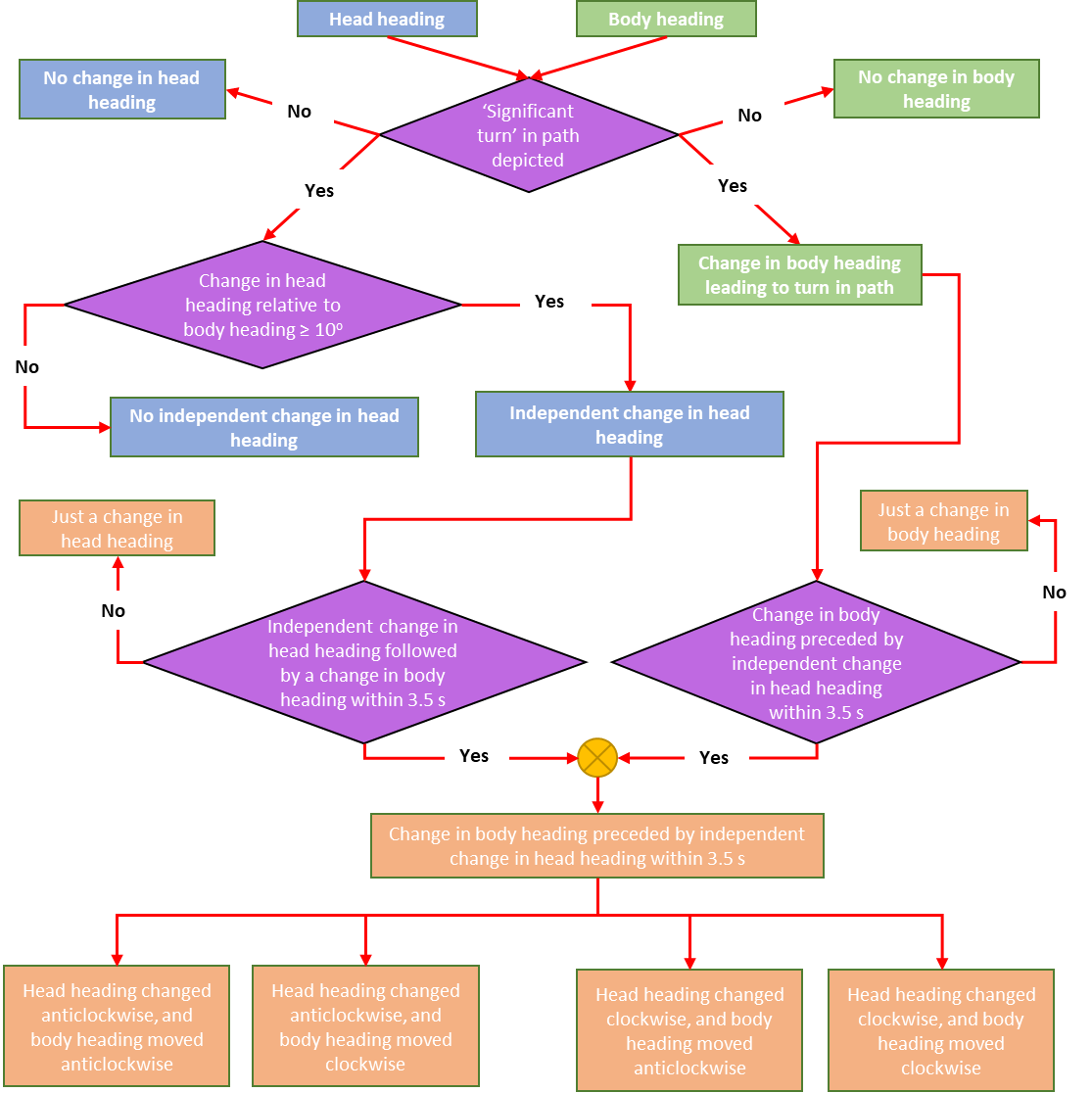


**Fig. S1. Schematic diagram of the how turns in oryx movement paths related to head heading.**


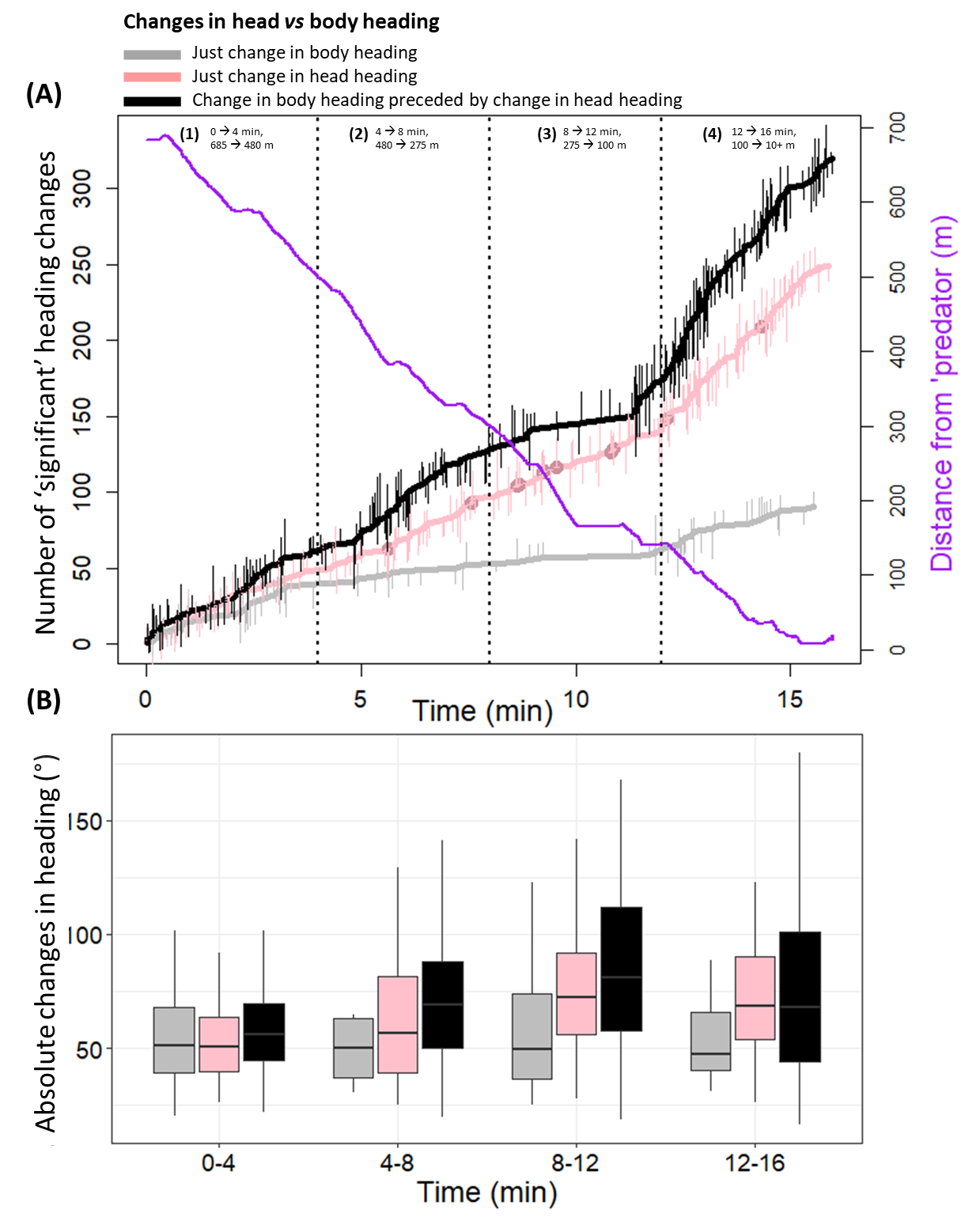


**Fig. S2. The relationship between the changes in (A) number and (B) magnitude of heading changes for 6 oryx in relation to the distance from an advancing person (referred to as the 'predator'; represented by the purple line in (A)).** [For further context, see Fig. 3A - single predator simulation, and animation S1]. In (A), pink circles superimposed on the pink line denoting 'just change in head heading' represent fixation periods, during which head heading remained unchanged for ≥ 2 s following a change in head heading. The length and direction of the vertical lines on the different types of heading change accumulation lines signify the extent and direction of the heading change with lines extending downwards corresponding to anti-clockwise heading changes, and lines extending upwards indicating clockwise changes. In (B), the boxes define the 25-75% interquartile range, the horizontal bars represent the median value, and whiskers extend to 1.5 times the interquartile range.
